# Psychopathological descriptive model of hallucinogenic/psychedelic drugs effect in the treatment of depression and addictions

**DOI:** 10.1192/j.eurpsy.2022.1820

**Published:** 2022-09-01

**Authors:** N. Girala

**Affiliations:** Consultorio, Psiquiatría, Asuncion, Paraguay

**Keywords:** Psychopathology, hallucinogenic drugs, psychedelic drugs, depression treatment

## Abstract

**Introduction:**

There is a growing and renewed interest in the use of hallucinogenic drugs in the treatment of psychiatric disorders, especially since the FDA approval of ketamine treatment for resistant depression. The response to hallucinogenic psychedelic substances (ayahuasca, psilocybin, LSD, ketamine) in the treatment of depression and addictions calls for a theoretical explanatory model.

**Objectives:**

Provide a descriptive / explanatory psychopathological model of the response to treatment with hallucinogenic drugs based on the descriptions of the subjects and the comparison with other extreme life experiences.

**Methods:**

Relevant published literature on subjective experiences in treatment with hallucinogenic drugs for depression and addictions is reviewed. It is compared with subjective experiences in life changing experiences.

**Results:**

Intense emotional states, mystical-type experiences including feelings of oneness, transcendence, ineffability, and the complex emotion of awe seem to be consistently presented as psychic elements related to the efficacy of these treatments. The genetic and cultural (memetic) evolutionary value of these emotions in the cohesiveness of human groups and the genesis of affective symptoms, and in the recalibration of cognitions and emotions, is discussed.

**Conclusions:**

The efficacy of hallucinogenic drugs used in the treatment of depression and addictions is accompanied by complex and varied emotions but with common psychopathological elements that could mediate their action.

**Disclosure:**

No significant relationships.

